# Carpal tunnel volume distribution and morphology changes with flexion-extension and radial-ulnar deviation wrist postures

**DOI:** 10.1371/journal.pone.0277234

**Published:** 2022-11-30

**Authors:** Drew A. Anderson, Michele L. Oliver, Karen D. Gordon

**Affiliations:** School of Engineering, University of Guelph, Guelph, Ontario, Canada; AIIMS: All India Institute of Medical Sciences, INDIA

## Abstract

Non-neutral wrist postures have been reported to cause decreased carpal tunnel volume (CTV) contributing to impingement of the median nerve and development of carpal tunnel syndrome. Recent analysis found CTV did not change with ±20° flexion-extension (FE), however, CTV decreased with ulnar deviation over the range of -5° to 15° radial-ulnar deviation (RUD). These findings suggest CTV may be too coarse of a measure to reflect the effects of slight non-neutral postures, or that volume is conserved and redistributed due to changes in tunnel morphology with posture. The objective of this study was to assess volume distribution along the length of the carpal tunnel and to quantify regional morphology changes with deviated wrist postures in both FE and RUD. Analysis was performed on a dataset of computed tomography scans collected on ten cadaveric specimens (5 male, 5 female, mean age = 80.7 ± 10.9 years) over a range of FE and RUD postures. The carpal tunnel of each scan was divided into four quartiles of equal length along the tunnel to quantify volume distribution. Volume within the carpal tunnel was seen to redistribute with both FE and RUD. Decreased volume in the distal aspect of the tunnel with flexion and proximal aspect of the tunnel with ulnar deviation may contribute to localized compression of the medial nerve. Measures of mean cross-sectional area, width and depth by quartile provided an indication of the morphology changes associated volume redistribution. Morphology analysis also revealed twisting between the proximal and distal aspects of the tunnel which increased with flexion and ulnar deviation and may further contribute to strain on the median nerve.

## Introduction

Carpal tunnel syndrome (CTS) is a debilitating neuropathy for which the aetiology is not fully understood. One mechanism that has been reported to lead to CTS is increased pressure within the carpal tunnel which causes compression on the median nerve [1]. Non-neutral wrist postures are a commonly reported risk factor for CTS, and have been reported to cause elevated carpal tunnel pressure [2, 3]. Due to the inverse relationship between pressure and volume, increased carpal tunnel pressure has long been thought to result from decreased carpal tunnel volume (CTV) [4–7]. Previous studies reported CTV to decrease with 30° of wrist flexion and extension relative to neutral posture [5–7]. However, a recent analysis focused on slight deviations from neutral wrist posture found that CTV did not significantly change over ± 20° range of flexion-extension (FE) [8]. Interestingly, a significant CTV decrease was observed between –5° and 15° of radial-ulnar deviation (RUD) [8]. Radial-ulnar deviation has been less studied with respect to CTV changes, with the focus on flexion and extension potentially due to patient reported symptoms or an emphasis on wrist FE in ergonomic assessment. These recent findings illustrate that further investigation of RUD is warranted and that, with slight changes in posture, the volume of the entire tunnel may be too course a measure to detect changes. Alternatively, changes in carpal tunnel morphology with posture may cause a redistribution of volume rather than a change in overall CTV. Despite no change in CTV, these morphology changes could produce localized pressure elevation, and thus mechanical impingement of the median nerve.

This study proposes a methodology to assess the how the carpal tunnel volume is distributed. Assessing volume distribution coupled with carpal tunnel morphology measures may provide novel insights on CTS etiology. Previous studies have quantified carpal morphology by performing measures on tunnel cross-sections with commonly reported metrics including cross-sectional area (CSA), width, and depth [9–13]. Recent work proposed novel metrics to assess carpal tunnel morphology using one-dimensional shape signatures of carpal tunnel cross sections taken along the length of the tunnel [14]. Capturing tunnel morphology in detail, these methods augment traditional methods for the assessment of carpal tunnel morphology. The objective of this study was to assess volume distribution along the length of the carpal tunnel and to quantify regional morphology changes with deviated wrist postures in both flexion-extension and radial-ulnar deviation.

## Methods

### Data collection

Analysis was performed on a computed tomography dataset of ten cadaveric specimens, (5 male, 5 female, mean age = 80.7 ± 10.9 years) [8]. The specimens were procured from Science Care Inc. (Phoenix, AZ, USA) where consent was obtained from the donors. The study was approved by the University of Guelph Research Ethics Board (REB# 16-12-786). Each specimen was imaged in target postures of −20°, −10°, −5°, 0°, 5°, 10°, and 20° of flexion with 0° of RUD, as well as −10°, −5°, 0°, 5°, 10° and 15° of RUD with 0° of FE. Precise FE and RUD angles of each posture were measured using inertia-based coordinate systems of the third metacarpal and the distal portion of the radius captured in the scan [15]. Three-dimensional carpal tunnel surface meshes with proximal and distal boundaries were generated for each scan based on previously described methods using anatomical landmarks of the transverse carpal ligament (TCL) [8, 16].

### Volume analysis

**Fig 1:**
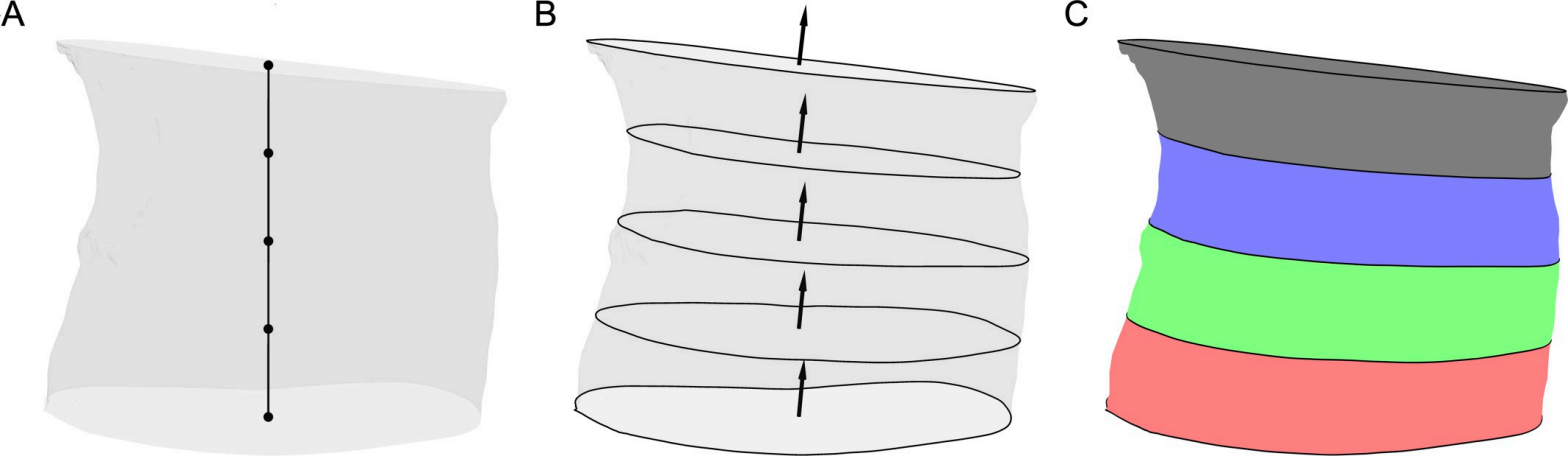
Division of carpal tunnel into quartiles to calculate volume distribution. Panel A shows points at 0%, 25%, 50%, 75%, and 100% of the distance along the vector between the centroids of the proximal and distal ends of a representative carpal tunnel in neutral posture. Panel B shows the planes which divide the tunnel oriented as a weighted average of the proximal and distal boundary normal vectors. Panel C shows the division of the tunnel volume into quartiles, where Q1(proximal) is red, Q2 is green, Q3 is blue, and Q4 (distal) is black.

To quantify CTV distribution, each carpal tunnel surface mesh was divided into four quartiles of equal length. Planes at 25%, 50%, and 75%, of the distance between the proximal and distal tunnel boundaries were defined to divide the tunnel into quartiles. The origin of each plane was defined as a percentage along the distance of a vector between the centroids of the proximal and distal tunnel cross-sections (Fig 1A). Orientation of the planes was defined as a weighted average of the proximal and distal boundary normal vectors (Fig 1B). Volume in mm^3^ was calculated for each of the four quartiles: Q1 (proximal-25%), Q2 (25–50%), Q3 (50–75%), and Q4 (75%-distal) (Fig 1C). Since the quartiles were defined in 25% sections based on the length of the tunnel, a change in overall tunnel length would affect each quartile equally. That is, a change in the length which could affect the total volume was equally distributed amongst the four quartiles.

### Morphology analysis

**Fig 2:**
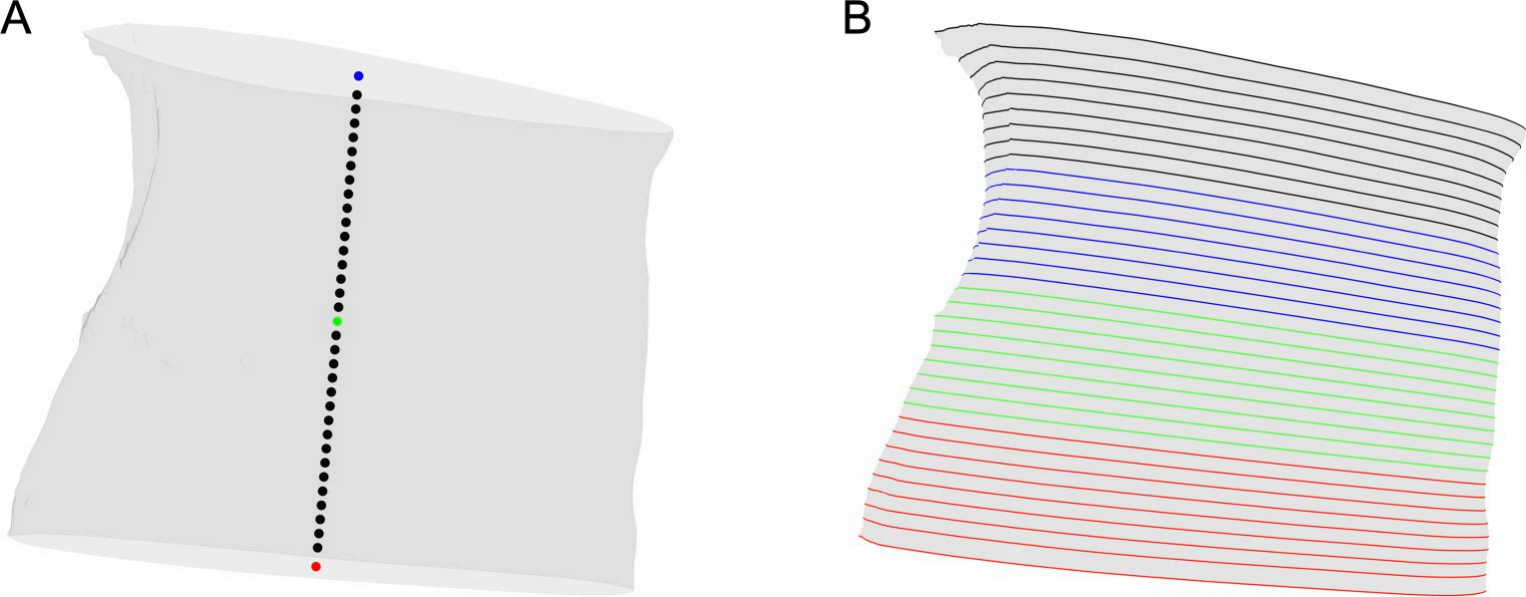
Carpal tunnel cross-sections used to calculate morphology metrics. Panel A shows the centroids of the cross-sections spaced at 0.5 mm increments starting from the midpoint (green) of the vector between the centroids of the proximal (red) and distal (blue) boundaries. Panel B shows the cross-sections of a representative carpal tunnel in neutral posture colour coded by volume quartile, where Q1(proximal) is red, Q2 is green, Q3 is blue, and Q4 (distal) is black.

Cross-sections were calculated at 0.5 mm intervals along the length of the tunnel. The centroids of the cross-sections were defined as points spaced 0.5 mm apart along a vector between the centroids of the proximal and distal carpal tunnel boundaries. The points were centered on the midpoint of the vector and the centroids of the proximal and distal carpal tunnel boundaries were defined as the first and last points respectively (Fig 2A). To accommodate for the length of each tunnel not being evenly divisible by 0.5 mm, the last interval between cross-sections at each end of the tunnel varied slightly with a mean distance of 0.53 ± 0.14 mm. The orientation of the cross-sections was defined as a weighted average of the proximal and distal tunnel boundaries (Fig 2B).

**Fig 3:**
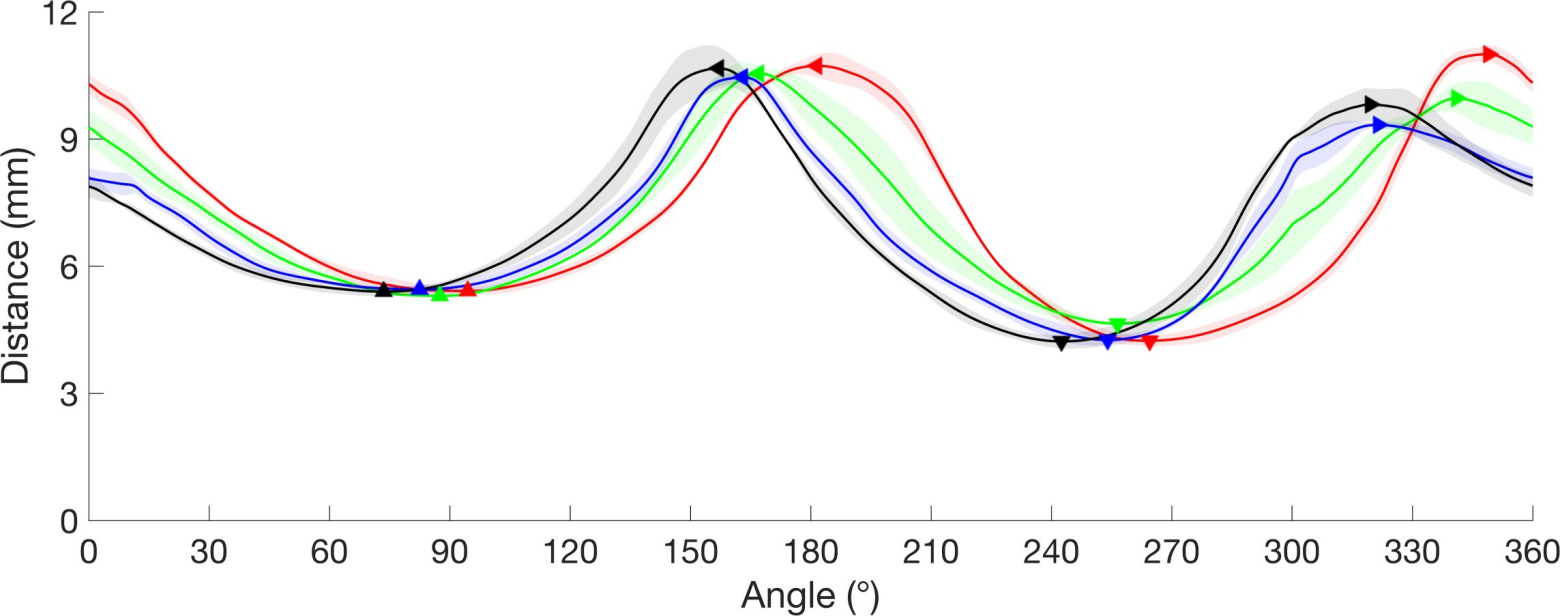
Quartile shape signatures of a representative carpal tunnel in neutral posture. The ensemble average ± standard deviation (shaded area) of the centroid-to-boundary distance shape signatures within quartiles where Q1 (proximal) is red, Q2 is green, Q3 is blue, and Q4 (distal) is black. Carpal tunnel width and depth were calculated as the summation of the medial (▶) and lateral (◀) peaks, and the palmar (▲) and dorsal (▼) troughs, respectively.

Several morphology measures were made on each cross-section and quartile averages were calculated for each metric. The morphology metrics in this study were determined using one-dimensional centroid-to-boundary distance shape signatures of each cross-section. For detailed description of the shape signature methodology please see [14]. In both [14] as well as the current study, a template shape signature was defined as a specimen specific size-normalized ellipse. The phase shift of each shape signature was calculated relative to the template with circular cross-correlation representing “twisting” of a cross-section relative to the template. The Euclidean distance from the template (EDT) was calculated for each cross-section shape signature with the phase shift removed. Larger distance values indicate greater deviation from the template shape. The cross-sectional area (CSA) was calculated by taking the integral of the shape signature. The width of the tunnel was calculated as the sum of the medial and lateral peaks of the shape signature. The depth of the tunnel was calculated as the sum of the palmar and dorsal peaks of the shape signature. Ensemble average shape signatures for a representative specimen in neutral posture are shown in Fig 3.

### Statistical analysis

Statistical analysis in this study was performed using R version 4.0.4 (R Foundation for Statistical Computing, Vienna, Austria). The effects of FE angle, RUD angle and the interaction between FE and RUD angle on carpal tunnel length were assessed by performing a linear regression with α = 0.05. Multiple linear regression was performed on quartile volume and each morphology metric at an α = 0.05 level of significance. Specimen was included as a categorical factor to account for anatomical variability between specimens. Scan order was included as a factor for the volume, CSA, width, and depth measures since the scans were collected in the same order for each specimen, and scan order was found to be a significant factor in a previous analysis of CTV [8]. Quartile was assessed as a main effect along with the first (linear) and second order (quadratic) effects of FE and RUD angle (FE, RUD, FE^2^, and RUD^2^). The two-way interactions between quartile and the angle terms were also included (Q:FE, Q:RUD, Q:FE^2^, and Q:RUD^2^). Each regression was performed using the full model with the second order and interaction terms included. If any of the second order or interaction terms were not significant, they were removed using a backward stepwise approach. Tukey honestly significant difference was used to determine the minimum significant difference between quartiles for α = 0.05.

## Results

**Fig 4:**
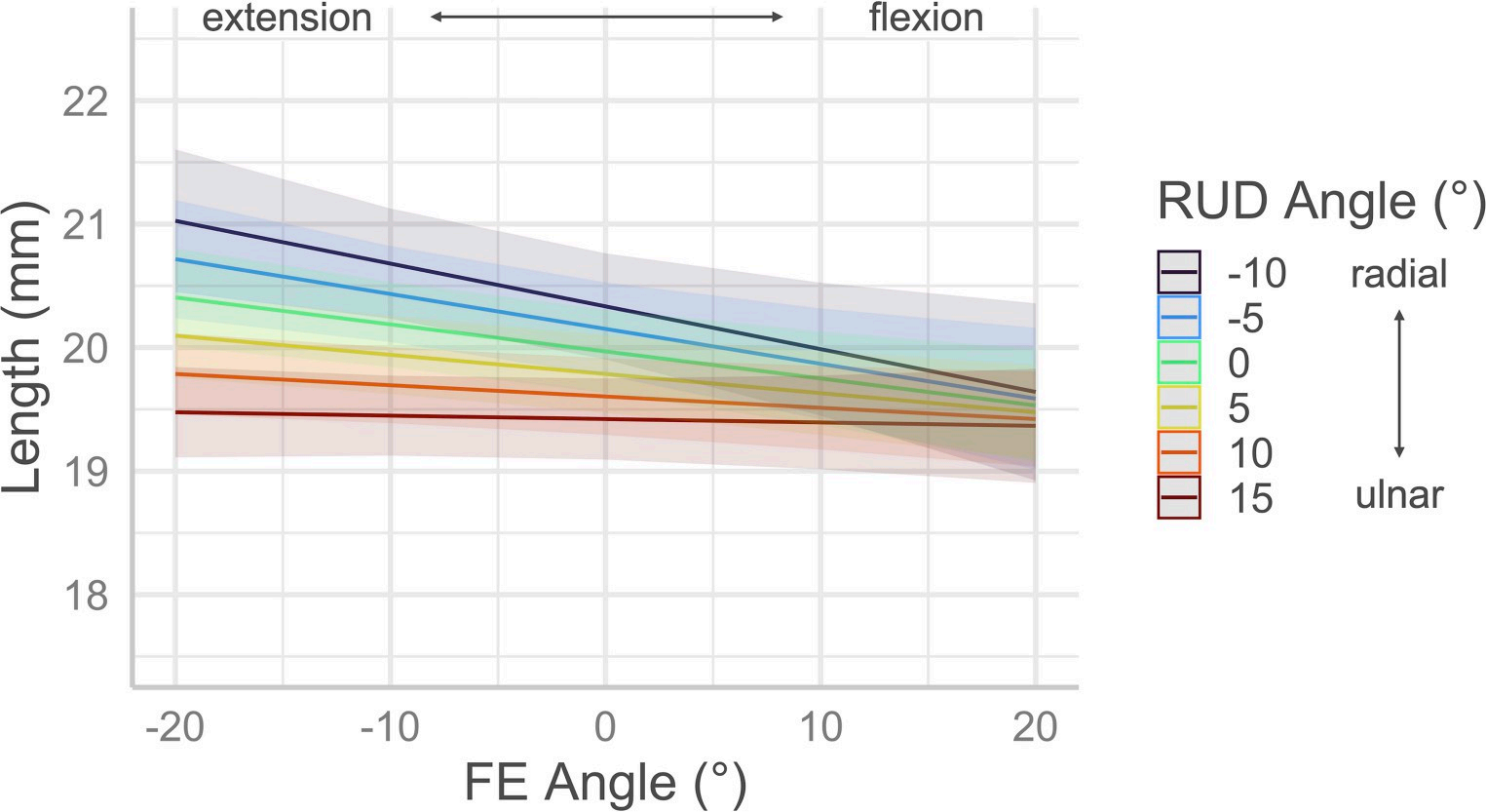
Effects of FE and RUD wrist postures on carpal tunnel length. The length of the carpal tunnel, as measured by the distance between the centroids of the proximal and distal cross-sections, decreases with flexion as well as with ulnar deviation. Shaded areas indicate 95% confidence intervals.

Regression analysis performed on carpal tunnel length revealed FE angle (*p* = 0.006) and RUD angle (*p* < 0.001) to be significant factors, while the FE RUD interaction approached significance (*p* = 0.06) with an overall model R^2^ of 0.95. The length of the tunnel decreased at an approximate rate of 0.02 mm per degree of FE with 0° of RUD, and at a rate of 0.04 mm per degree of RUD with 0° of FE. The effects of combined FE and RUD on tunnel length are demonstrated in Fig 4 which was generated using the based on the regression model with the interaction term included.

**Fig 5:**
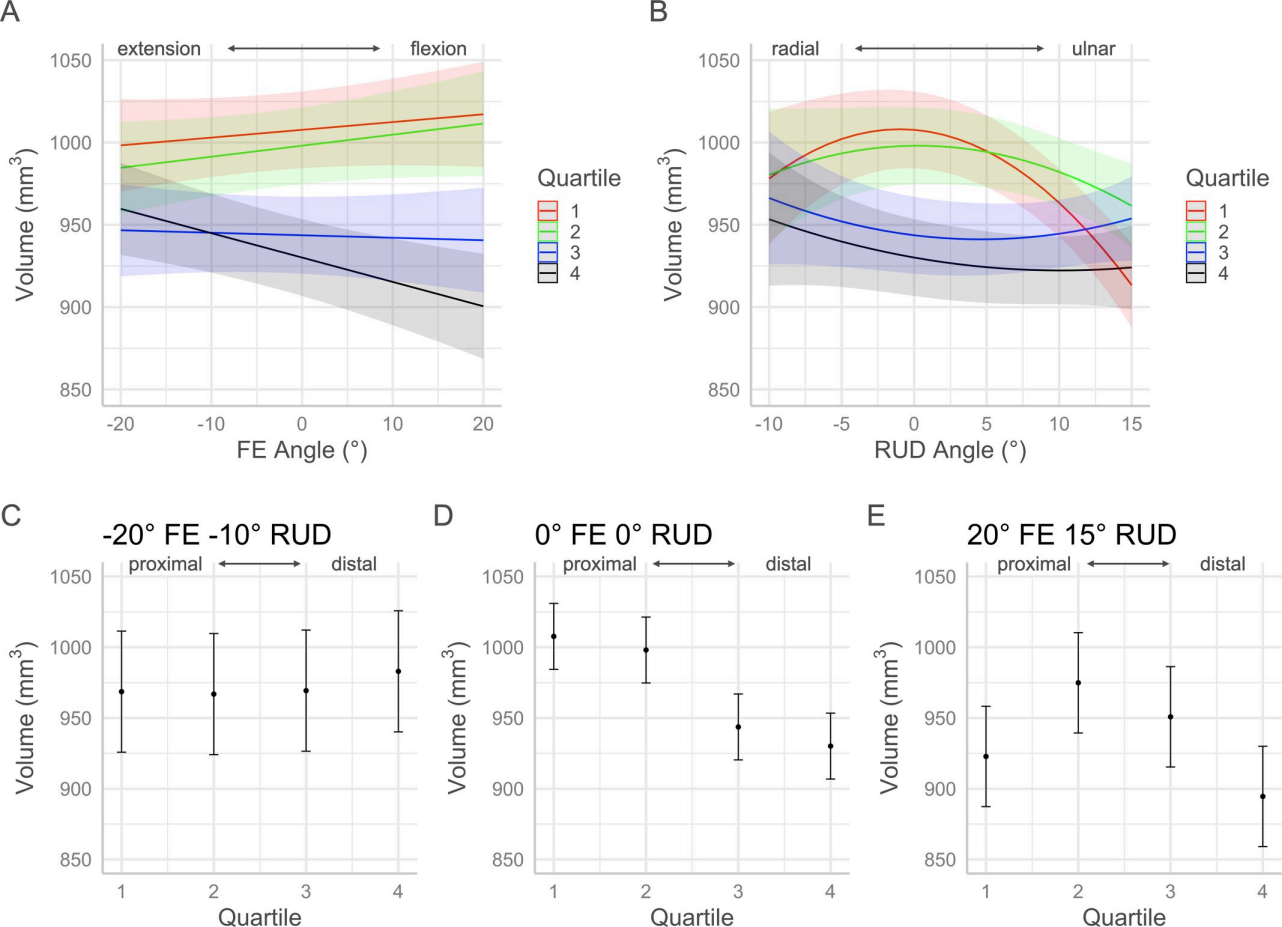
Effects of FE and RUD wrist postures on carpal tunnel volume distribution by quartile. Panel A shows the effects of varying FE angle with 0° RUD. Panel B shows the effect of varying RUD angle with 0° FE. Panels C/D/E show the predicted values with 95% confidence intervals for each quartile for an extended radially deviated posture (panel C), a neutral posture (panel D), and a flexed and ulnarly deviated posture (panel E).

**Fig 6:**
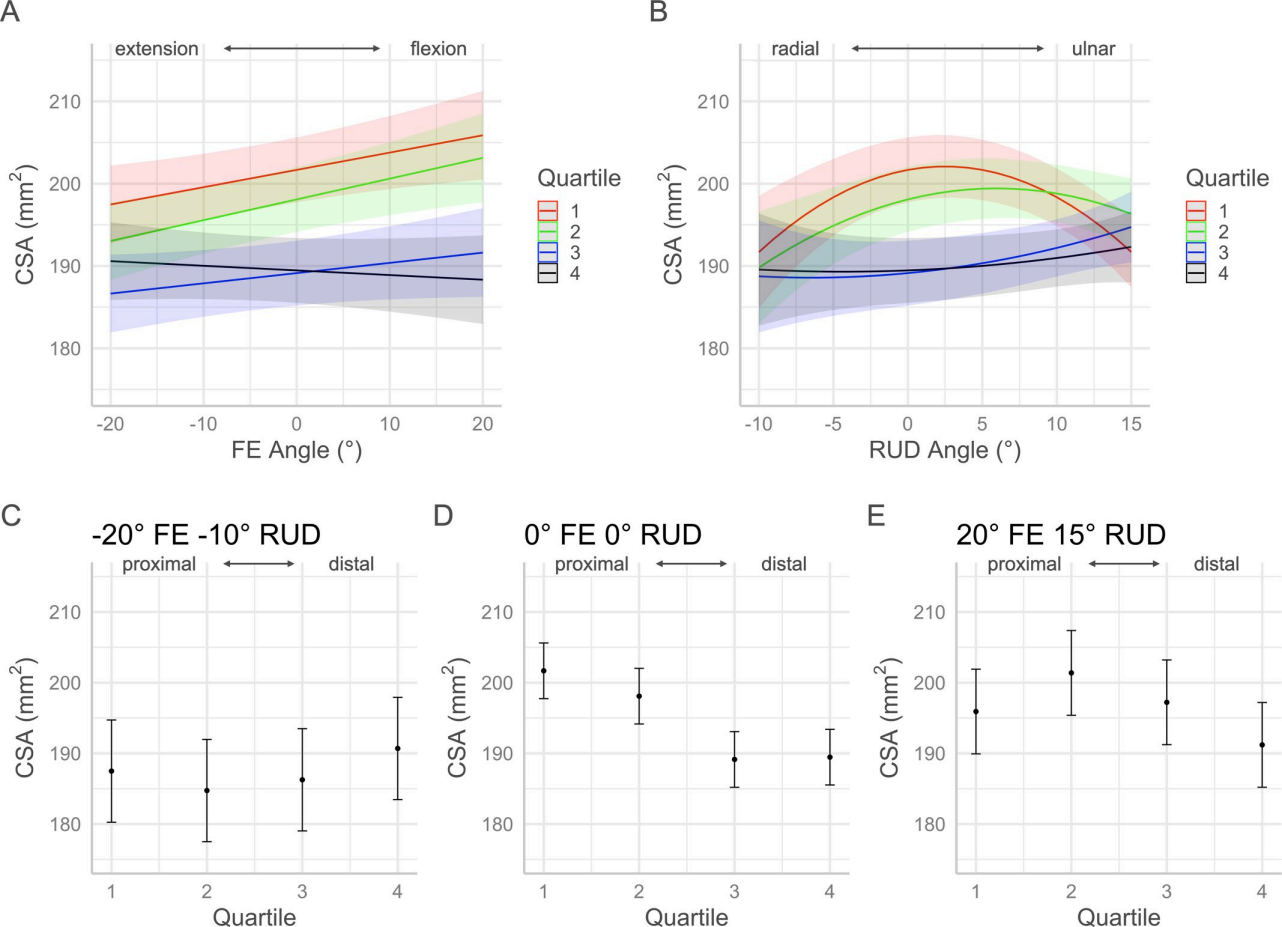
Effects of FE and RUD wrist postures on the mean cross-sectional area within each quartile. Panel A shows the effects of varying FE angle with 0° RUD. Panel B shows the effect of varying RUD angle with 0° FE. Panels C/D/E show the predicted values with 95% confidence intervals for each quartile for an extended radially deviated posture (panel C), a neutral posture (panel D), and a flexed and ulnarly deviated posture (panel E).

**Fig 7:**
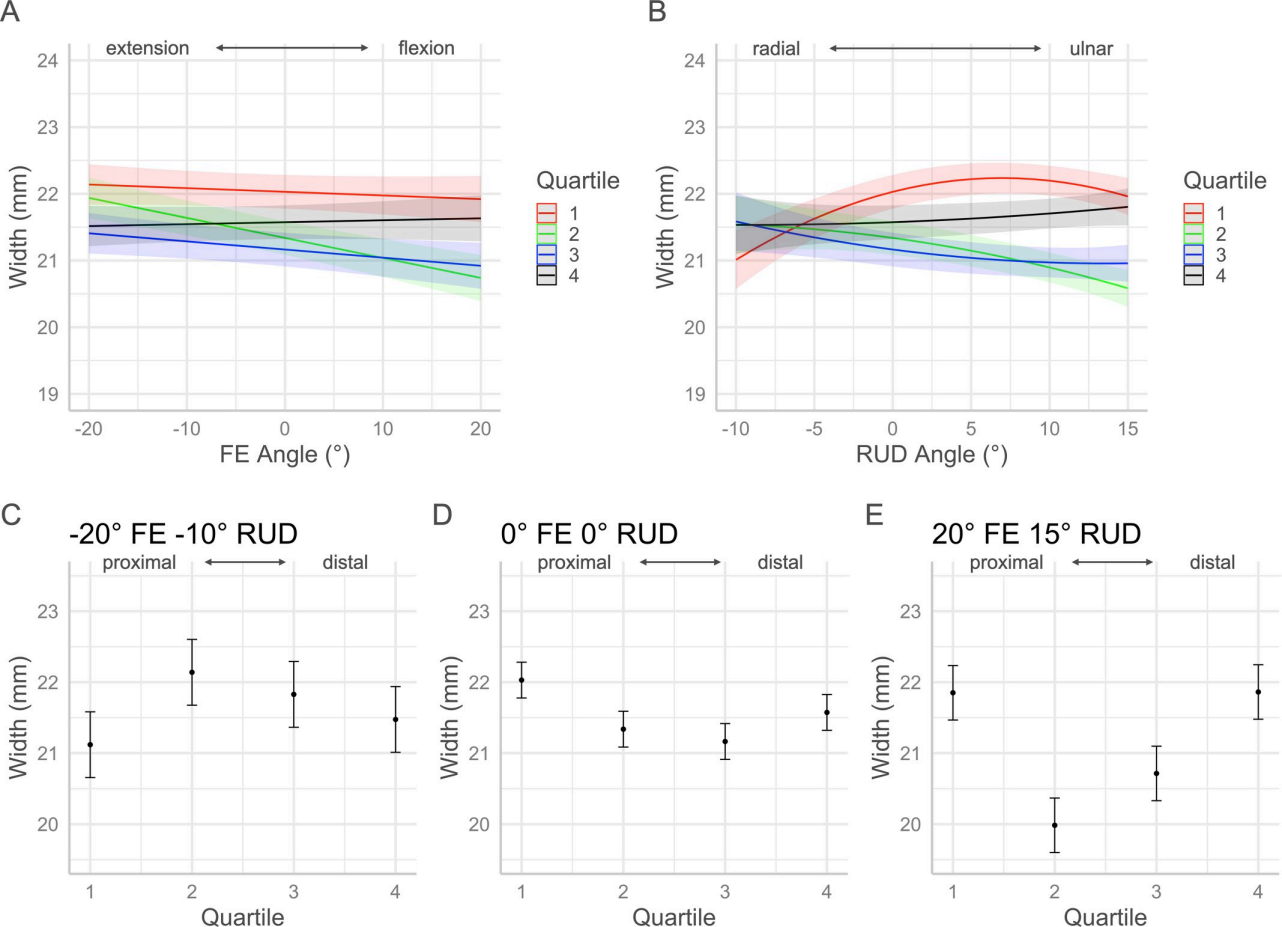
Effects of FE and RUD wrist postures on the mean carpal tunnel width within each quartile. Panel A shows the effects of varying FE angle with 0° RUD. Panel B shows the effect of varying RUD angle with 0° FE. Panels C/D/E show the predicted values with 95% confidence intervals for each quartile for an extended radially deviated posture (panel C), a neutral posture (panel D), and a flexed and ulnarly deviated posture (panel E).

**Fig 8:**
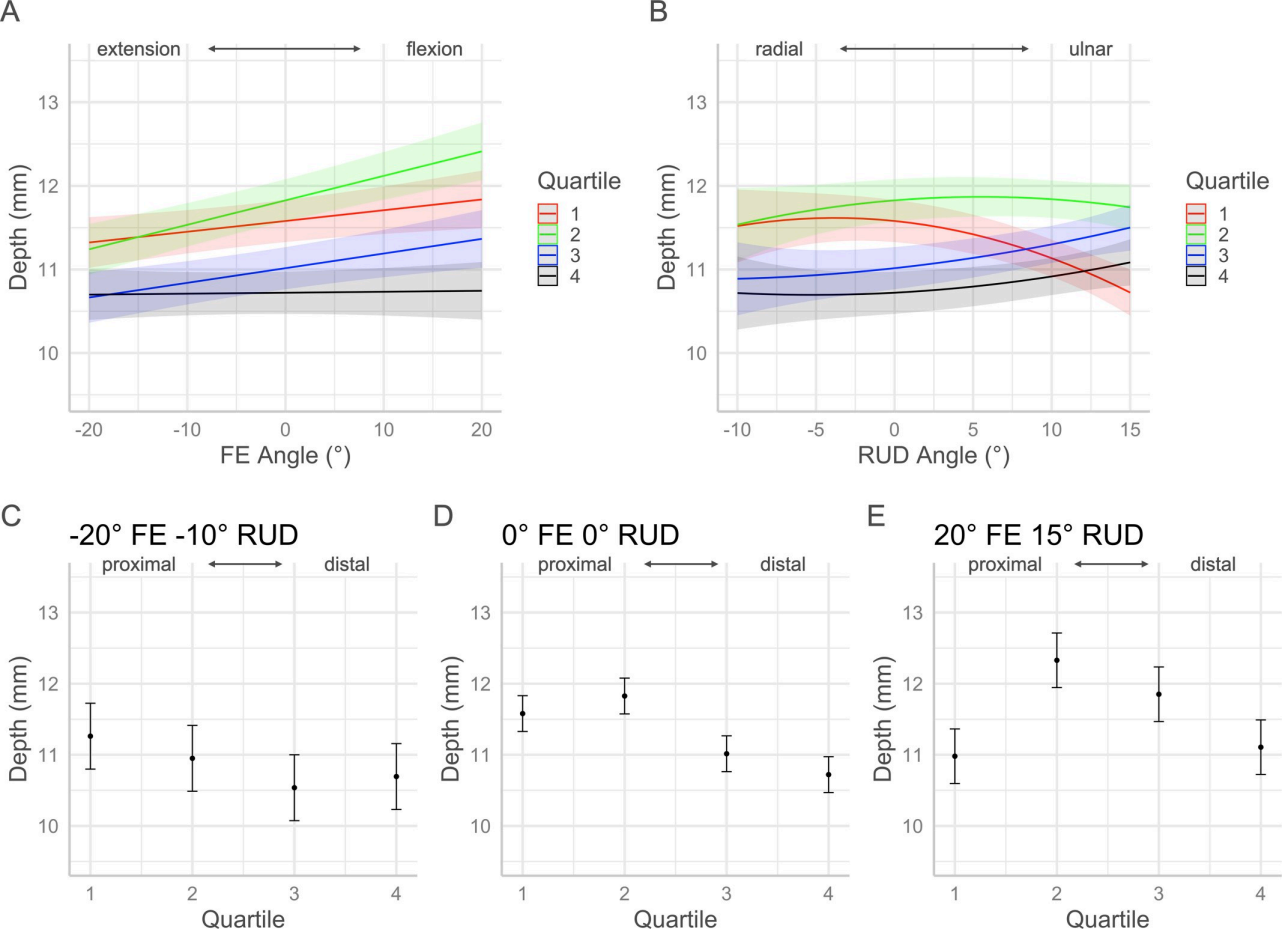
Effects of FE and RUD wrist postures on the mean carpal tunnel depth within each quartile. Panel A shows the effects of varying FE angle with 0° RUD. Panel B shows the effect of varying RUD angle with 0° FE. Panels C/D/E show the predicted values with 95% confidence intervals for each quartile for an extended radially deviated posture (panel C), a neutral posture (panel D), and a flexed and ulnarly deviated posture (panel E).

**Fig 9:**
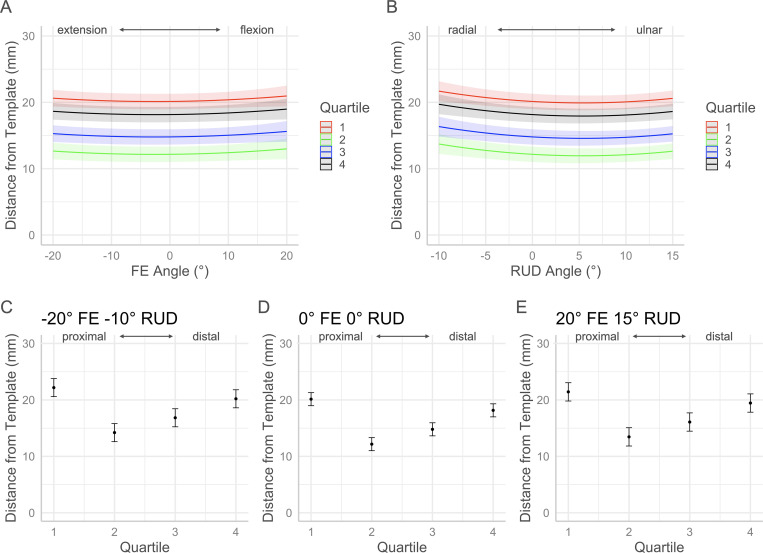
Effects of FE and RUD wrist postures on the mean shape signature distance from the template within each quartile. Panel A shows the effects of varying FE angle with 0° RUD. Panel B shows the effect of varying RUD angle with 0° FE. Panels C/D/E show the predicted values with 95% confidence intervals for each quartile for an extended radially deviated posture (panel C), a neutral posture (panel D), and a flexed and ulnarly deviated posture (panel E).

**Fig 10:**
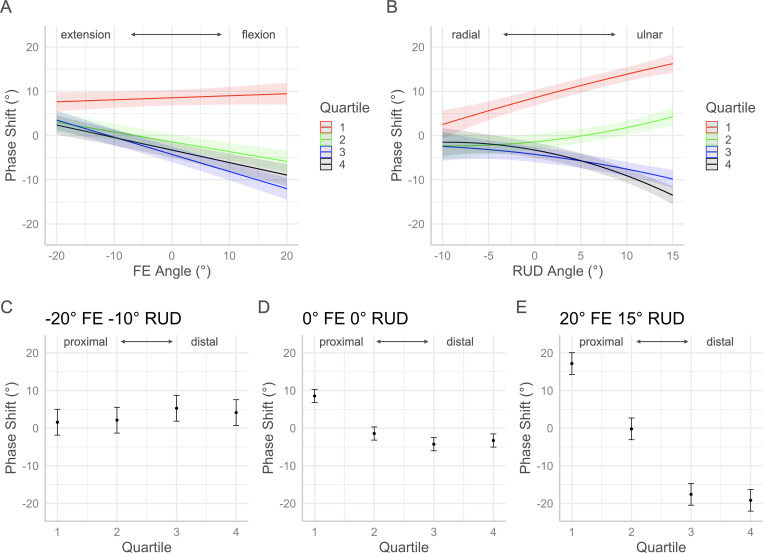
Effects of FE and RUD wrist postures on the mean shape signature phase shift within each quartile. Panel A shows the effects of varying FE angle with 0° RUD. Panel B shows the effect of varying RUD angle with 0° FE. Panels C/D/E show the predicted values with 95% confidence intervals for each quartile for an extended radially deviated posture (panel C), a neutral posture (panel D), and a flexed and ulnarly deviated posture (panel E).

The *p*-values for each factor of the volume and morphology full regression models are contained in Table 1. Interaction plots with 95% confidence intervals in Figs 5–10 show the effects by quartile of ±20° of FE with 0° of RUD (panel A) and -10° to 15° RUD with 0° of FE (panel B). These panels show the predicted effect of pure FE and pure RUD over the range of postures tested in this study. Panels C-E of Figs 5–10 show the predicted values by quartile with 95% confidence intervals for an extended and radially deviated posture (-20° FE, -10° RUD) in panel C, neutral posture (0° FE, 0° RUD) in panel D, and a flexed and ulnarly deviated posture (20° FE, 15° RUD) in panel E. These panels demonstrate the effects of combined FE and RUD postures relative to neutral. The posture combinations were selected because the wrist tends toward radial deviation with extension and ulnar deviation with flexion [17].

**Table 1:** P-values for each factor in the full multiple regression models of volume, cross-sectional area (CSA), width, depth, Euclidean distance from the template (EDT), and phase shift relative to the template.

	P–Value					
	Volume	CSA	Width	Depth	EDT	Phase Shift
**Specimen**	< 0.001	< 0.001	< 0.001	< 0.001	< 0.001	< 0.001
**Scan order**	< 0.001	< 0.001	0.04	< 0.001	0.24^a^	< 0.001
**Quartile**	< 0.001	< 0.001	< 0.001	< 0.001	< 0.001	< 0.001
**Radial-Ulnar (RUD)**	0.001	0.30	0.33	0.95	0.59	0.47
**Flexion-Extension (FE)**	0.99	0.001	0.001	< 0.001	0.10	< 0.001
**RUD^2^**	0.057	0.007	0.046	0.26	0.009	0.18
**FE^2^**	0.41^a^	0.27^a^	0.17^a^	0.21^a^	0.02	0.51^a^
**Quartile:RUD**	0.001	0.018	< 0.001	< 0.001	0.94^b^	< 0.001
**Quartile:FE**	0.003	0.019	< 0.001	< 0.001	0.11^b^	< 0.001
**Quartile:RUD^2^**	< 0.001	< 0.001	< 0.001	0.008	0.14^b^	0.007
**Quartile:FE^2^**	0.96^b^	0.79^b^	0.59^b^	0.96^b^	0.98^b^	0.98^b^
**R^2^ Value^c^**	0.96	0.95	0.92	0.85	0.77	0.79

^**a**^ Terms which were not significant and that were not in a significant second order or interaction term were removed

^**b**^ Interaction terms which were not significant were removed

^**c**^ R^**2**^ Values of the final regression models with non-significant factors (denoted by ^**a**^ and ^**b**^) omitted

The minimum significant volume difference between quartiles across all postures was 19.15 mm^3^. In neutral posture (0° FE, 0° RUD), the volumes of Q1 and Q2 were significantly greater than Q3 and Q4 (Fig 5D). The regression coefficients used to generate the plots in Fig 5A and 5B are provided in Eq 1. The total CTV for a given posture can be predicted by the summation of the predicted quartile volumes calculated with Eq 1. The predicted CTV in postures of -20°, 0°, and 20° of FE with 0° of RUD was 3895 mm^3^, 3885 mm^3^, and 3875 mm^3^ respectively. The predicted CTV in postures of -10°, 0°, and 15° of RUD with 0° of FE was 3883 mm^3^, 3885 mm^3^, and 3758 mm^3^. The changes in carpal tunnel morphology which occur along with changes in volume distribution are seen in Figs 6–10.


$$volume(Q,\theta_{RUD},\theta_{FE})=\left\{ \begin{array}{l} -0.37\;{\theta_{RUD}}^{2}-0.74\;\theta_{RUD}+0.47\;\theta_{FE}+1009,\;Q=1\\ -0.17\;{\theta_{RUD}}^{2}+0.091\;\theta_{RUD}+0.67\;\theta_{FE}+999,\;Q=2\\ \;0.12\;{\theta_{RUD}}^{2}-1.1\;\theta_{RUD}-0.15\;\theta_{FE}+945,\;Q=3\\ \;0.077\;{\theta_{RUD}}^{2}-1.6\;\theta_{RUD}-1.5\;\theta_{FE}+931,\;Q=4 \end{array}\right.$$
Eq 1: Regression equations for volume by quartile

## Discussion

### Volume distribution

This study is the first to evaluate distribution of carpal tunnel volume with changing wrist posture. Volume and morphology changes along the length of the tunnel were detected by dividing the carpal tunnel in each posture into quartiles, four sections of equal length. While the tunnel could be divided into any number of arbitrary sub-sections, quartiles were chosen to provide granularity to changes occurring at the most proximal (Q1) and distal (Q4) aspects of the tunnel relative to the middle (Q2 and Q3) of the tunnel. However, for volume and the majority of the morphology metrics, the proximal quartiles (Q1 and Q2) and the distal quartiles (Q3 and Q4) behaved similarly and the trends can be described with the tunnel into proximal and distal halves. In neutral posture, quartile volume decreased proximally to distally with the proximal half (Q1 and Q2) being significantly larger than the distal half (Q3 and Q4). Moving through the range of 20° extension to 20° flexion, the volume of the proximal half increased while the distal half decreased. The greatest effect was seen with the most distal quartile Q4, which decreased at a rate of 1.6 mm^3^/degree, corresponding with a decrease of approximately 7% over the ±20° range of FE. Previous analysis of the same dataset found that the total CTV did not change over the ±20° range of motion [8]. Therefore, total CTV was preserved as the wrist flexed through a range of ±20° of FE but was redistributed from the distal half to the proximal half of the tunnel. This volume decrease in the distal region of the carpal tunnel with flexion may cause compression of the median nerve despite the overall volume not changing.

Over the range of -10° to 15° of RUD, the proximal half of the tunnel volume behaved as negative quadratics with a maximum at approximately 0° of RUD (Fig 5). Conversely, the distal half behaved as positive quadratics with a minimum volume between 5° and 10° of ulnar deviation. The greatest effect was seen for the most proximal quartile (Q1) which had the largest quartile volume in neutral and the smallest quartile volume with 15° of ulnar deviation. For both radial and ulnar deviation, the volume in the proximal half of the tunnel decreased relative to neutral while the volume in the distal half of the tunnel increased relative to neutral. The effect of these trends was that the total CTV decreased with ulnar deviation. This result supports previous analysis of the same dataset where overall CTV was seen to decrease at a rate of −5.9 mm^3^/degree from radial to ulnar deviation [8]. The findings of the present study indicate that the reduction in CTV with ulnar deviation is driven by the volume in the proximal region decreasing more than the volume increasing in the distal half of the tunnel. Furthermore, the effect of ulnar deviation on median nerve compression may be greater than expected due to the total CTV decrease as well as the concentrated decrease in the proximal region of the tunnel.

Further insight on the distribution of CTV can be gained from examining combined FE and RUD postures (Fig 5). The effect of FE on volume was the greatest in the distal half of the tunnel, particularly on Q4 (most distal), while the effect of RUD on volume was the greatest on the proximal half of the tunnel particularly on Q1 (most proximal). The result of these trends is that for a posture of -20° FE and -10° RUD (Fig 5C), the volume of the proximal half decreased while the volume of the distal half increased relative to neutral. In this case, the total CTV of the extended and radially deviated posture is preserved relative to the volume in neutral posture. While neutral posture is often considered most favourable with respect to CTS risk, this finding suggests that slight wrist extension combined with slight radial deviation may not be any worse in a clinical context. In contrast, the effects of combined flexion and ulnar deviation result in a reduction in the overall CTV. For a posture of 20° FE and 15° RUD (Fig 5E), the volume in both the proximal and distal halves of the tunnel decreased relative to neutral. This result was driven by large reductions in volume in the most proximal quartile (Q1) as well as the most distal quartile (Q4). Therefore, the combination of slight flexion and slight radial deviation caused volume restriction at each end of the tunnel which could be put an individual at risk for developing or exacerbating CTS. Reduced volume in a quartile could contribute to evaluated pressure in a localized area or mechanical impingement of the median nerve. Future study which quantifies median nerve deformation in addition to carpal tunnel volume distribution is warranted to further improve our understanding of CTS etiology and risk factors.

### Cross-sectional area

The morphology analysis performed in this study provides insight into the factors contributing to carpal tunnel volume redistribution with posture. Since volume is equal to area multiplied by length, examining the mean CSA in each quartile in combination with the length of the tunnel can explain the trends seen for volume distribution. Over the ±20° range of FE assessed in this study, the mean CSA in the proximal half of the tunnel (Q1 and Q2) increased with flexion. The CSA in Q3 increased with flexion as well, but at a lesser rate than Q1 and Q2, while the CSA in Q4 (the most distal quartile) decreased with flexion. Over the range of ±20° of FE the total length of the tunnel decreased by 0.88 mm. The increase in CSA with flexion in the proximal half of the tunnel was large enough to cause a volume increase despite the decrease in tunnel length. Although the CSA in Q3 increased, the decreased tunnel length resulted in a slight decrease in Q3 volume. The effect of decreased Q4 CSA with flexion was amplified by the decreased tunnel length leading to the decrease in Q4 volume. The result of these trends was that moving from 20° of extension through to 20° of flexion, the tunnel got shorter while the CSA increased distally to proximally. The decrease in CSA at the most distal aspect of the tunnel could indicate a localized compression with flexion, despite the overall volume not changing significantly over the range of ±20° FE.

Over the range of -10° to 15° of RUD, the trends for the mean CSA of each quartile were similar to the trends in quartile volume; the proximal half of the tunnel (Q1 & Q2) behaved as negative quadratics while the distal half of the tunnel (Q3 & Q4) behaved as positive quadratics. The CSA in the proximal half of the tunnel was greatest with slight ulnar deviation but decreased with radial deviation and further ulnar deviation. The CSA in the distal half of the tunnel was smallest with slight radial deviation but increased with ulnar deviation. At the ends of the range of RUD, the mean CSA was relatively equivalent between the four quartiles. Over the range of -10° to 15° of RUD the total length of the tunnel decreased linearly by 0.91 mm. While the trends in CSA suggest that the volume should increase in ulnar compared to radial deviation, the opposite occurred due to the decrease in tunnel length with ulnar deviation.

For the combination of -20° of extension with -10° of radial deviation, the CSA in the proximal half of the tunnel decreased significantly relative to neutral while the CSA in the distal half remained approximately the same as in neutral. This posture coincided with the longest tunnel length which lessened the effect of decreased CSA in the proximal half on the volume. With the combination of 20° of flexion and 15° of ulnar deviation, the CSA at the ends of the tunnel, Q1 and Q4, were lower than the middle of the tunnel, Q2 and Q3. The constriction at the ends of the tunnel coupled with the decrease in tunnel length with flexion and ulnar deviation, meant that volume at the ends of the tunnel was the lowest with the posture of combined flexion and ulnar deviation.

### Width and depth

The width and depth of the tunnel are two factors which represent shape changes in the carpal tunnel which correspond to changes in CSA. Over the range of ± 20° FE, the mean width (medial-lateral distance) changed very little in the most proximal (Q1) and most distal (Q4) quartiles. The small changes in Q1 and Q4 tunnel width with FE are comparable to the amount of medial-lateral translation of the scaphoid relative to the pisiform, and the trapezium relative to the hamate reported by Moojen et al. in their analysis of carpal kinematics with FE [18]. Therefore, carpal tunnel width at the proximal and distal ends of the carpal tunnel is constrained by the low medial-lateral translation within the carpal rows during FE motion. With RUD, the width of the proximal region (Q1) decreased with radial deviation and increased with ulnar deviation relative to neutral. The width of the tunnel in Q4 increased slightly with ulnar deviation but did not change with radial deviation. These results are consistent with the greater intercarpal bone motion reported by Moojen et al. within the proximal carpal row than the distal carpal row with RUD [18]. The mean width in the middle of the tunnel (Q2 & Q3) decreased from extension to flexion as well as from radial to ulnar deviation. In contrast, the mean depth of the tunnel increased from extension to flexion as well as from radial to ulnar deviation. Relative to neutral, the implications of the opposite trends of width and depth suggest that the shape of the tunnel middle is more rounded (circular) with flexion, ulnar deviation, and combined flexion with ulnar deviation. Conversely, the shape of the tunnel middle is flattened (elliptical) with extension, radial deviation, and combined extension and radial deviation. The more rounded shape corresponds with increased CSA and volume in the middle section of the tunnel, whereas the flatter shape corresponds with decreased CSA and volume in the middle section of the tunnel. The width and depth in the most proximal region of the tunnel (Q1) follow a similar trend as the middle of the tunnel with FE, however, with RUD the width increased with ulnar deviation while the depth decreased with ulnar deviation. Therefore, moving from radial to ulnar deviation the proximal end of the tunnel is flattening which corresponds with a decreased volume in Q1. These results could indicate that with ulnar deviation, the median nerve is subject to increased compression where the nerve enters the carpal tunnel. The most distal aspect of the tunnel (Q4) had the lowest mean depth across the range of FE, while the width did not vary greatly. As a result, the distal aspect of the tunnel remained relatively flat with flexion compared to the other regions of the tunnel contributing to a lower volume in Q4.

### Shape signature morphology

Analysis of carpal tunnel shape signatures was performed to quantify the mean Euclidean distance and phase shift of the cross-sections in each quartile relative to a template function. The template function was defined for each specimen as an ellipse with major and minor axes equal to the width and depth of the middle of the tunnel in neutral posture. This definition of the template function was developed with the purpose of detecting shape changes along the length of the tunnel with changes in posture [14]. Used to detect shape changes, the EDT measure was found to be sensitive to twisting along the tunnel length, therefore, the phase shift of each shape signature relative to the template was removed prior to calculating EDT [14]. Across all postures, the EDT was greatest in the most proximal region of the tunnel (Q1) followed by the most distal region (Q4). EDT increased slightly relative to neutral in all quartiles with extension, flexion, and radial deviation. These results indicate that the ends of the tunnel vary in shape from the middle of the tunnel, but the shape in each quartile does not change noticeably with posture. Detailed morphology of the tunnel at the proximal, middle, and distal levels for approximately neutral posture was described in a recent analysis of the same dataset [14].

**Fig 11:**
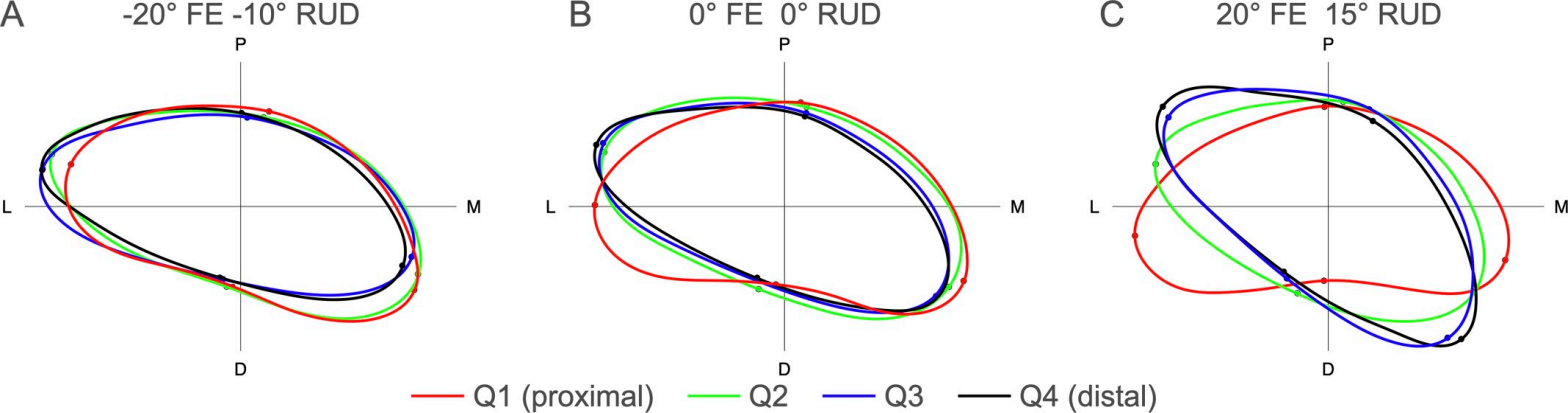
Illustration of the predicted mean (n = 10) twist along the carpal tunnel in neutral and with combined FE and RUD postures. Panel A demonstrates 20° extension with 10° of radial deviation where Q3 (blue) and Q4 (black) exhibit slight external rotation relative to Q1 (red) and Q2 (green). Panel B demonstrates neutral posture (0° FE and 0° RUD) where Q2, Q3, and Q4 are internally rotated relative to Q1. Panel C demonstrates 20° flexion with 15° of ulnar deviation where Q2 is internally rotated relative to Q1 and Q3 and Q4 are further internally rotated relative to Q2 and Q1.

The phase shift relative to the template describes twisting along the length of the tunnel with the baseline being the orientation of the middle of the tunnel in neutral posture. In neutral posture, the mean phase shift for Q1 relative to the template is approximately 9° whereas the mean phase shift for Q4 relative to the template is approximately -4°. This result indicates there was approximately 13° of internal rotation of the distal region of the tunnel relative to the proximal region. Over the range of FE, Q1 rotates externally, while Q2, Q3, and Q4 rotate internally from extension through to flexion. In -20° FE, the tunnel is relatively aligned along the length but is slightly externally rotated relative to neutral. In 20° of FE there is nearly 20° of internal rotation of the distal region relative to the proximal region. Similar trends occur with RUD where the proximal half of the tunnel rotates externally, and the distal half of the tunnel rotates internally with ulnar deviation. The tunnel becomes roughly aligned in -10° RUD but there is nearly 30° of twist between the proximal and distal regions with 15° RUD. In the combined posture of -20° FE and -10° RUD, the tunnel is relatively aligned with the distal half of the tunnel slightly externally rotated (< 5°) relative to the proximal half of the tunnel. With 20° FE and 15° of RUD the distal half of the tunnel is internally rotated by approximately 35° relative to the proximal region of the tunnel. Fig 11 demonstrates the effects of combined FE and RUD on the twist along the tunnel through representative illustrations of the mean contours across all of the specimens within each quartile. The illustrations were constructed with the predicted medial, lateral, palmar, and dorsal points for each posture determined with methods described in [14].

This study is the first to report twisting along the length of the carpal tunnel with changes in wrist posture. As such, the clinical significance of this twisting along the length of the carpal tunnel is unknown, therefore, further analysis assessing twisting with CTS severity in a clinical population is warranted. The internal rotation of the distal region relative to the proximal region with flexion and ulnar deviation was correlated with decreased tunnel length indicating a “wringing” action. Twisting along the length of the tunnel could contribute to compression of the median nerve or increase shear loading on the subsynovial connective tissues (SSCT) of the tendons that pass though the carpal tunnel. The SSCT serves to facilitate gliding of the finger flexor tendons within the carpal tunnel and also has the biomechanical function to absorb and transmit stress [19]. Shear stresses on the SSCT associated with repetitive and high force movements of the hand are thought to contribute to the pathophysiology of CTS [20]. In addition, the gliding resistance of the flexor digitorum superficialis tendons within the carpal tunnel has been reported to increase with wrist flexion compared to neutral and extension postures [21]. Twist between the proximal and distal aspects of the tunnel in wrist flexion may affect the structure of the SSCT and be a potential explanation for increased gliding resistance with wrist flexion.

### Limitations

The limitations of this study should be considered when interpreting the results. With respect to volume distribution, analysis was performed by dividing the tunnel arbitrarily into quartiles based on the total tunnel length. Therefore, this method assumes the total length of the tunnel in each posture to be evenly divided amongst the quartiles. In reality, the tunnel may not deform evenly over the entire length. However, this assumption was necessary in the absence of detectible anatomical landmarks of carpal tunnel soft tissues in the computed tomography dataset. Another limitation is that the morphology metrics were reported as the mean value within each quartile. While quartile means permitted comparison of morphology trends with quartile volume trends, some resolution may be lost, particularly in assessing the morphology of the most proximal and most distal aspects of the tunnel. The range of postures assessed in this study is an additional limitation. The dataset of computed tomography images was collected with a focus on slight deviations from neutral posture. Therefore, the findings are limited to the ±20° range of FE and -10° to 15° range of RUD postures.

## Conclusions

This study demonstrated carpal tunnel volume redistribution with both FE and RUD, providing additional insight on the role posture plays in CTS pathophysiology. While the total CTV was not seen to vary with slight flexion and extension, decreased volume in the distal aspect of the tunnel with flexion may contribute to localized compression of the medial nerve. Total CTV decreased with ulnar deviation which was driven by the volume reduction in the proximal aspect of the tunnel, again pointing to the potential for local median nerve compression. Taken together, these trends indicate that combined flexion and ulnar deviation postures should be avoided as volume was seen to decrease in both the proximal and distal aspects of the tunnel, even in slight non-neutral postures. Assessing the mean CSA, width and depth along the tunnel, in addition to the length of the tunnel provided an indication of the shape changes and associated volume redistribution. In addition, novel analysis using a shape signature representation of the carpal tunnel revealed a twisting action whereby the distal aspect of the tunnel rotated internally relative to the proximal aspect of the tunnel with flexion as well as with ulnar deviation. Although the clinical implications are uncertain, twisting along the length of the carpal tunnel, coupled with the other morphology changes with posture, could induce strain on the median nerve and exacerbate symptoms of CTS. Based on the findings of this study, clinicians and ergonomists should ensure that FE, RUD, and combined FE and RUD wrist postures are considered in CTS treatment and prevention. Future study which compares a CTS and control group using the methods presented in the present study may provide further insight on the clinical significance of volume distribution and the associated morphology changes with deviated wrist postures.

## Supporting information

S1 Table Regression tables for each outcome variable. Regression output from R for each outcome variable with 95% confidence intervals for each regression coefficient. The categorical factor for specimen is denoted by sfac and the categorical factor for quartile is denoted by qfac.
(DOCX)

S1 File Minimal dataset. File contains all data required to replicate the study findings including the reported statistical tests and the figures in the results.
(XLSX)
