# Supplementary material for: Carpal tunnel volume distribution and morphology changes with flexion-extension and radial-ulnar deviation wrist postures
Source: PLoS One. 2022 Nov 30;17(11):e0277234. doi: 10.1371/journal.pone.0277234 (PMC9710750; doi:10.1371/journal.pone.0277234)
Supplement: S1 Table — Regression output from R for each outcome variable with 95% confidence intervals for each regression coefficient. The categorical factor for specimen is denoted by sfac and the categorical factor for quartile is denoted by qfac. (DOCX) [file pone.0277234.s001.docx]

| Length | | | | | | |
| --- | --- | --- | --- | --- | --- | --- |
|  | **Estimate** | **2.5 %** | **97.5 %** | **Std.Error** | **p value** |  |
| (Intercept) | 17.410 | 17.094 | 17.726 | 0.159 | 2.00E-16 | *** |
| sfac2 | 2.218 | 1.777 | 2.659 | 0.222 | 2.00E-16 | *** |
| sfac3 | 2.559 | 2.108 | 3.010 | 0.227 | 2.00E-16 | *** |
| sfac4 | 5.281 | 4.855 | 5.707 | 0.215 | 2.00E-16 | *** |
| sfac5 | 0.809 | 0.389 | 1.228 | 0.211 | 0.000233 | *** |
| sfac6 | 3.215 | 2.732 | 3.698 | 0.243 | 2.00E-16 | *** |
| sfac7 | 6.211 | 5.782 | 6.640 | 0.216 | 2.00E-16 | *** |
| sfac8 | 0.108 | -0.335 | 0.552 | 0.223 | 0.628597 |  |
| sfac9 | 0.951 | 0.488 | 1.414 | 0.233 | 9.33E-05 | *** |
| sfac10 | 1.327 | 0.898 | 1.756 | 0.216 | 1.92E-08 | *** |
| RUD_angle | -0.036 | -0.053 | -0.020 | 0.008 | 3.43E-05 | *** |
| FE_angle | -0.022 | -0.035 | -0.009 | 0.007 | 0.00141 | ** |
| RUD_angle:FE_angle | 0.001 | 0.000 | 0.003 | 0.001 | 0.058742 | . |
|  |  |  |  |  |  |  |
| Signif. codes: 0 ‘***’ 0.001 ‘**’ 0.01 ‘*’ 0.05 ‘.’ 0.1 ‘ ’ 1 | | | | | |  |
|  | | | | | |  |
| Residual standard error: 0.4909 on 95 degrees of freedom | | | | | |  |
| Multiple R-squared: 0.9513, Adjusted R-squared: 0.9452 | | | | | |  |
| F-statistic: 154.7 on 12 and 95 DF, p-value: < 2.2e-16 | | | | | |  |

| Volume | | | | | | |
| --- | --- | --- | --- | --- | --- | --- |
|  | **Estimate** | **2.5 %** | **97.5 %** | **Std.Error** | **p value** |  |
| (Intercept) | 605.498 | 576.211 | 634.785 | 14.898 | 2.00E-16 | *** |
| sfac2 | 261.799 | 237.444 | 286.154 | 12.389 | 2.00E-16 | *** |
| sfac3 | 344.129 | 319.157 | 369.101 | 12.703 | 2.00E-16 | *** |
| sfac4 | 591.594 | 567.890 | 615.298 | 12.058 | 2.00E-16 | *** |
| sfac5 | 118.779 | 95.458 | 142.100 | 11.863 | 2.00E-16 | *** |
| sfac6 | 305.604 | 278.301 | 332.907 | 13.889 | 2.00E-16 | *** |
| sfac7 | 900.449 | 876.407 | 924.491 | 12.230 | 2.00E-16 | *** |
| sfac8 | 119.732 | 95.045 | 144.418 | 12.558 | 2.00E-16 | *** |
| sfac9 | 554.801 | 529.221 | 580.380 | 13.012 | 2.00E-16 | *** |
| sfac10 | 78.031 | 53.526 | 102.535 | 12.465 | 9.81E-10 | *** |
| scan_order_abs | 5.808 | 3.992 | 7.624 | 0.924 | 8.33E-10 | *** |
| qfac2 | -9.633 | -31.832 | 12.566 | 11.292 | 0.39412 |  |
| qfac3 | -64.031 | -86.230 | -41.832 | 11.292 | 2.71E-08 | *** |
| qfac4 | -77.576 | -99.775 | -55.377 | 11.292 | 2.43E-11 | *** |
| RUD_angle | -0.736 | -2.774 | 1.302 | 1.037 | 0.47797 |  |
| FE_angle | 0.471 | -0.474 | 1.417 | 0.481 | 0.32755 |  |
| I(RUD_angle^2) | -0.370 | -0.530 | -0.210 | 0.081 | 7.15E-06 | *** |
| qfac2:RUD_angle | 0.828 | -1.979 | 3.634 | 1.428 | 0.5624 |  |
| qfac3:RUD_angle | -0.350 | -3.156 | 2.457 | 1.428 | 0.80657 |  |
| qfac4:RUD_angle | -0.820 | -3.626 | 1.987 | 1.428 | 0.56621 |  |
| qfac2:FE_angle | 0.198 | -1.040 | 1.437 | 0.630 | 0.7532 |  |
| qfac3:FE_angle | -0.623 | -1.862 | 0.615 | 0.630 | 0.32317 |  |
| qfac4:FE_angle | -1.951 | -3.190 | -0.713 | 0.630 | 0.00209 | ** |
| qfac2:I(RUD_angle^2) | 0.201 | -0.017 | 0.420 | 0.111 | 0.07002 | . |
| qfac3:I(RUD_angle^2) | 0.488 | 0.270 | 0.706 | 0.111 | 1.39E-05 | *** |
| qfac4:I(RUD_angle^2) | 0.447 | 0.229 | 0.665 | 0.111 | 6.61E-05 | *** |
|  |  |  |  |  |  |  |
| Signif. codes: 0 ‘***’ 0.001 ‘**’ 0.01 ‘*’ 0.05 ‘.’ 0.1 ‘ ’ 1 | | | | | | |
|  | | | | | | |
| Residual standard error: 54.84 on 406 degrees of freedom | | | | | | |
| Multiple R-squared: 0.9629, Adjusted R-squared: 0.9606 | | | | | | |
| F-statistic: 421.7 on 25 and 406 DF, p-value: < 2.2e-16 | | | | | | |

| CSA | | | | | | |
| --- | --- | --- | --- | --- | --- | --- |
|  | **Estimate** | **2.5 %** | **97.5 %** | **Std.Error** | **p value** |  |
| (Intercept) | 140.078 | 135.134 | 145.023 | 2.51522 | 2.00E-16 | *** |
| sfac2 | 36.255 | 32.144 | 40.367 | 2.09161 | 2.00E-16 | *** |
| sfac3 | 51.561 | 47.345 | 55.777 | 2.14463 | 2.00E-16 | *** |
| sfac4 | 69.921 | 65.919 | 73.922 | 2.03575 | 2.00E-16 | *** |
| sfac5 | 21.044 | 17.107 | 24.982 | 2.00281 | 2.00E-16 | *** |
| sfac6 | 39.466 | 34.857 | 44.076 | 2.34479 | 2.00E-16 | *** |
| sfac7 | 115.244 | 111.185 | 119.303 | 2.06478 | 2.00E-16 | *** |
| sfac8 | 28.093 | 23.926 | 32.261 | 2.12011 | 2.00E-16 | *** |
| sfac9 | 115.102 | 110.783 | 119.420 | 2.19681 | 2.00E-16 | *** |
| sfac10 | 9.080 | 4.943 | 13.217 | 2.1045 | 2.01E-05 | *** |
| scan_order_abs | 1.004 | 0.697 | 1.310 | 0.15594 | 3.46E-10 | *** |
| qfac2 | -3.586 | -7.334 | 0.162 | 1.90648 | 0.06068 | . |
| qfac3 | -12.541 | -16.289 | -8.793 | 1.90648 | 1.47E-10 | *** |
| qfac4 | -12.214 | -15.962 | -8.466 | 1.90648 | 4.12E-10 | *** |
| RUD_angle | 0.333 | -0.011 | 0.677 | 0.17503 | 0.05774 | . |
| FE_angle | 0.210 | 0.051 | 0.370 | 0.08119 | 0.00994 | ** |
| I(RUD_angle^2) | -0.067 | -0.094 | -0.040 | 0.01374 | 1.84E-06 | *** |
| qfac2:RUD_angle | 0.118 | -0.356 | 0.592 | 0.24101 | 0.6252 |  |
| qfac3:RUD_angle | -0.161 | -0.634 | 0.313 | 0.24101 | 0.5053 |  |
| qfac4:RUD_angle | -0.263 | -0.737 | 0.211 | 0.24101 | 0.27598 |  |
| qfac2:FE_angle | 0.042 | -0.167 | 0.251 | 0.10638 | 0.69142 |  |
| qfac3:FE_angle | -0.086 | -0.295 | 0.123 | 0.10638 | 0.41937 |  |
| qfac4:FE_angle | -0.267 | -0.476 | -0.058 | 0.10638 | 0.01256 | * |
| qfac2:I(RUD_angle^2) | 0.029 | -0.008 | 0.065 | 0.01872 | 0.12734 |  |
| qfac3:I(RUD_angle^2) | 0.080 | 0.043 | 0.117 | 0.01872 | 2.48E-05 | *** |
| qfac4:I(RUD_angle^2) | 0.075 | 0.038 | 0.111 | 0.01872 | 8.10E-05 | *** |
|  |  |  |  |  |  |  |
| Signif. codes: 0 ‘***’ 0.001 ‘**’ 0.01 ‘*’ 0.05 ‘.’ 0.1 ‘ ’ 1 | | | | | | |
|  | | | | | | |
| Residual standard error: 9.258 on 406 degrees of freedom | | | | | | |
| Multiple R-squared: 0.9498, Adjusted R-squared: 0.9467 | | | | | | |
| F-statistic: 307 on 25 and 406 DF, p-value: < 2.2e-16 | | | | | | |

| Width | | | | | | |
| --- | --- | --- | --- | --- | --- | --- |
|  | **Estimate** | **2.5 %** | **97.5 %** | **Std.Error** | **p value** |  |
| (Intercept) | 20.057 | 19.740 | 20.374 | 0.161 | 2.00E-16 | *** |
| sfac2 | 1.125 | 0.861 | 1.388 | 0.134 | 8.03E-16 | *** |
| sfac3 | 1.614 | 1.344 | 1.884 | 0.137 | 2.00E-16 | *** |
| sfac4 | 5.020 | 4.763 | 5.276 | 0.130 | 2.00E-16 | *** |
| sfac5 | 1.558 | 1.306 | 1.811 | 0.128 | 2.00E-16 | *** |
| sfac6 | 3.232 | 2.936 | 3.527 | 0.150 | 2.00E-16 | *** |
| sfac7 | 6.033 | 5.773 | 6.293 | 0.132 | 2.00E-16 | *** |
| sfac8 | 0.898 | 0.631 | 1.165 | 0.136 | 1.20E-10 | *** |
| sfac9 | 4.274 | 3.997 | 4.551 | 0.141 | 2.00E-16 | *** |
| sfac10 | 0.750 | 0.485 | 1.015 | 0.135 | 4.82E-08 | *** |
| scan_order_abs | 0.036 | 0.016 | 0.056 | 0.010 | 0.000368 | *** |
| qfac2 | -0.691 | -0.932 | -0.451 | 0.122 | 2.88E-08 | *** |
| qfac3 | -0.865 | -1.105 | -0.625 | 0.122 | 6.33E-12 | *** |
| qfac4 | -0.456 | -0.696 | -0.216 | 0.122 | 0.000219 | *** |
| RUD_angle | 0.059 | 0.037 | 0.081 | 0.011 | 2.01E-07 | *** |
| FE_angle | -0.005 | -0.016 | 0.005 | 0.005 | 0.29364 |  |
| I(RUD_angle^2) | -0.004 | -0.006 | -0.003 | 0.001 | 1.83E-06 | *** |
| qfac2:RUD_angle | -0.092 | -0.122 | -0.061 | 0.015 | 6.63E-09 | *** |
| qfac3:RUD_angle | -0.090 | -0.120 | -0.060 | 0.015 | 1.13E-08 | *** |
| qfac4:RUD_angle | -0.051 | -0.081 | -0.020 | 0.015 | 0.001099 | ** |
| qfac2:FE_angle | -0.025 | -0.038 | -0.011 | 0.007 | 0.000362 | *** |
| qfac3:FE_angle | -0.007 | -0.020 | 0.007 | 0.007 | 0.325368 |  |
| qfac4:FE_angle | 0.008 | -0.005 | 0.022 | 0.007 | 0.219279 |  |
| qfac2:I(RUD_angle^2) | 0.003 | 0.001 | 0.005 | 0.001 | 0.011236 | * |
| qfac3:I(RUD_angle^2) | 0.005 | 0.003 | 0.008 | 0.001 | 9.08E-06 | *** |
| qfac4:I(RUD_angle^2) | 0.005 | 0.002 | 0.007 | 0.001 | 0.0001 | *** |
|  |  |  |  |  |  |  |
| Signif. codes: 0 ‘***’ 0.001 ‘**’ 0.01 ‘*’ 0.05 ‘.’ 0.1 ‘ ’ 1 | | | | | | |
|  |  |  |  |  |  |  |
| Residual standard error: 0.5934 on 406 degrees of freedom | | | | | | |
| Multiple R-squared: 0.9232, Adjusted R-squared: 0.9185 | | | | | | |
| F-statistic: 195.2 on 25 and 406 DF, p-value: < 2.2e-16 | | | | | | |

| Depth | | | | | | |
| --- | --- | --- | --- | --- | --- | --- |
|  | **Estimate** | **2.5 %** | **97.5 %** | **Std.Error** | **p value** |  |
| (Intercept) | 8.757 | 8.440 | 9.074 | 0.161 | 2.00E-16 | *** |
| sfac2 | 1.729 | 1.466 | 1.993 | 0.134 | 2.00E-16 | *** |
| sfac3 | 2.433 | 2.163 | 2.703 | 0.137 | 2.00E-16 | *** |
| sfac4 | 0.993 | 0.736 | 1.249 | 0.130 | 1.87E-13 | *** |
| sfac5 | 0.598 | 0.346 | 0.850 | 0.128 | 4.28E-06 | *** |
| sfac6 | 1.705 | 1.410 | 2.001 | 0.150 | 2.00E-16 | *** |
| sfac7 | 2.819 | 2.559 | 3.079 | 0.132 | 2.00E-16 | *** |
| sfac8 | 1.047 | 0.780 | 1.313 | 0.136 | 9.81E-14 | *** |
| sfac9 | 4.641 | 4.365 | 4.918 | 0.141 | 2.00E-16 | *** |
| sfac10 | 0.098 | -0.166 | 0.363 | 0.135 | 0.465552 |  |
| scan_order_abs | 0.039 | 0.019 | 0.059 | 0.010 | 0.000109 | *** |
| qfac2 | 0.247 | 0.007 | 0.487 | 0.122 | 0.043792 | * |
| qfac3 | -0.565 | -0.805 | -0.325 | 0.122 | 4.99E-06 | *** |
| qfac4 | -0.858 | -1.098 | -0.618 | 0.122 | 8.67E-12 | *** |
| RUD_angle | -0.019 | -0.041 | 0.003 | 0.011 | 0.087093 | . |
| FE_angle | 0.013 | 0.003 | 0.023 | 0.005 | 0.013722 | * |
| I(RUD_angle^2) | -0.003 | -0.004 | -0.001 | 0.001 | 0.004277 | ** |
| qfac2:RUD_angle | 0.034 | 0.004 | 0.065 | 0.015 | 0.026189 | * |
| qfac3:RUD_angle | 0.040 | 0.009 | 0.070 | 0.015 | 0.010264 | * |
| qfac4:RUD_angle | 0.029 | -0.001 | 0.059 | 0.015 | 0.059645 | . |
| qfac2:FE_angle | 0.016 | 0.003 | 0.030 | 0.007 | 0.016411 | * |
| qfac3:FE_angle | 0.005 | -0.009 | 0.018 | 0.007 | 0.4939 |  |
| qfac4:FE_angle | -0.012 | -0.025 | 0.002 | 0.007 | 0.085994 | . |
| qfac2:I(RUD_angle^2) | 0.001 | -0.001 | 0.004 | 0.001 | 0.340726 |  |
| qfac3:I(RUD_angle^2) | 0.003 | 0.001 | 0.006 | 0.001 | 0.005937 | ** |
| qfac4:I(RUD_angle^2) | 0.003 | 0.001 | 0.006 | 0.001 | 0.003911 | ** |
|  |  |  |  |  |  |  |
| Signif. codes: 0 ‘***’ 0.001 ‘**’ 0.01 ‘*’ 0.05 ‘.’ 0.1 ‘ ’ 1 | | | | | | |
|  |  |  |  |  |  |  |
| Residual standard error: 0.5928 on 406 degrees of freedom | | | | | | |
| Multiple R-squared: 0.8608, Adjusted R-squared: 0.8523 | | | | | | |
| F-statistic: 100.4 on 25 and 406 DF, p-value: < 2.2e-16 | | | | | | |

| Euclidean distance from the template (EDT) | | | | | | |
| --- | --- | --- | --- | --- | --- | --- |
|  | **Estimate** | **2.5 %** | **97.5 %** | **Std.Error** | **p value** |  |
| (Intercept) | 15.727 | 14.110 | 17.344 | 0.823 | 2.00E-16 | *** |
| sfac2 | 2.827 | 1.495 | 4.160 | 0.678 | 3.69E-05 | *** |
| sfac3 | 3.402 | 2.034 | 4.769 | 0.695 | 1.44E-06 | *** |
| sfac4 | 8.369 | 7.073 | 9.666 | 0.660 | 2.00E-16 | *** |
| sfac5 | -0.684 | -1.960 | 0.592 | 0.649 | 0.2929 |  |
| sfac6 | 10.772 | 9.278 | 12.265 | 0.760 | 2.00E-16 | *** |
| sfac7 | 10.047 | 8.732 | 11.363 | 0.669 | 2.00E-16 | *** |
| sfac8 | 2.820 | 1.467 | 4.172 | 0.688 | 4.99E-05 | *** |
| sfac9 | 11.915 | 10.514 | 13.316 | 0.713 | 2.00E-16 | *** |
| sfac10 | 0.676 | -0.666 | 2.018 | 0.683 | 0.3227 |  |
| scan_order_abs | 0.090 | -0.015 | 0.196 | 0.054 | 0.0939 | . |
| qfac2 | -7.971 | -8.773 | -7.168 | 0.408 | 2.00E-16 | *** |
| qfac3 | -5.343 | -6.145 | -4.540 | 0.408 | 2.00E-16 | *** |
| qfac4 | -1.979 | -2.782 | -1.176 | 0.408 | 1.77E-06 | *** |
| RUD_angle | -0.067 | -0.127 | -0.008 | 0.031 | 0.0275 | * |
| FE_angle | -0.001 | -0.035 | 0.032 | 0.017 | 0.9347 |  |
| I(RUD_angle^2) | 0.006 | 0.002 | 0.011 | 0.002 | 0.0099 | ** |
| I(FE_angle^2) | 0.002 | 0.000 | 0.004 | 0.001 | 0.0206 | * |
|  |  |  |  |  |  |  |
| Signif. codes: 0 ‘***’ 0.001 ‘**’ 0.01 ‘*’ 0.05 ‘.’ 0.1 ‘ ’ 1 | | | | | | |
|  |  |  |  |  |  |  |
| Residual standard error: 3 on 414 degrees of freedom | | | | | | |
| Multiple R-squared: 0.7748, Adjusted R-squared: 0.7656 | | | | | | |
| F-statistic: 83.8 on 17 and 414 DF, p-value: < 2.2e-16 | | | | | | |

| Phase Shift | | | | | | |
| --- | --- | --- | --- | --- | --- | --- |
|  | **Estimate** | **2.5 %** | **97.5 %** | **Std.Error** | **p value** |  |
| (Intercept) | 7.570 | 5.172 | 9.969 | 1.220 | 1.34E-09 | *** |
| sfac2 | 5.372 | 3.378 | 7.366 | 1.014 | 1.95E-07 | *** |
| sfac3 | 4.560 | 2.515 | 6.604 | 1.040 | 1.49E-05 | *** |
| sfac4 | 0.970 | -0.971 | 2.910 | 0.987 | 0.32674 |  |
| sfac5 | 0.809 | -1.100 | 2.719 | 0.971 | 0.40527 |  |
| sfac6 | -0.903 | -3.138 | 1.333 | 1.137 | 0.42774 |  |
| sfac7 | 5.376 | 3.407 | 7.344 | 1.001 | 1.34E-07 | *** |
| sfac8 | -0.164 | -2.185 | 1.858 | 1.028 | 0.87358 |  |
| sfac9 | 0.047 | -2.047 | 2.142 | 1.065 | 0.96462 |  |
| sfac10 | -2.960 | -4.967 | -0.954 | 1.021 | 0.00393 | ** |
| scan_order_abs | 0.000 | -0.149 | 0.148 | 0.076 | 0.99525 |  |
| qfac2 | -9.965 | -11.782 | -8.147 | 0.925 | 2.00E-16 | *** |
| qfac3 | -12.800 | -14.621 | -10.986 | 0.925 | 2.00E-16 | *** |
| qfac4 | -11.820 | -13.642 | -10.007 | 0.925 | 2.00E-16 | *** |
| RUD_angle | 0.569 | 0.402 | 0.736 | 0.085 | 7.04E-11 | *** |
| FE_angle | 0.046 | -0.032 | 0.123 | 0.039 | 0.24559 |  |
| I(RUD_angle^2) | -0.004 | -0.017 | 0.010 | 0.007 | 0.58992 |  |
| qfac2:RUD_angle | -0.364 | -0.594 | -0.134 | 0.117 | 0.00197 | ** |
| qfac3:RUD_angle | -0.825 | -1.055 | -0.595 | 0.117 | 7.42E-12 | *** |
| qfac4:RUD_angle | -0.947 | -1.177 | -0.717 | 0.117 | 6.40E-15 | *** |
| qfac2:FE_angle | -0.268 | -0.369 | -0.166 | 0.052 | 3.36E-07 | *** |
| qfac3:FE_angle | -0.434 | -0.535 | -0.332 | 0.052 | 7.29E-16 | *** |
| qfac4:FE_angle | -0.329 | -0.430 | -0.227 | 0.052 | 5.01E-10 | *** |
| qfac2:I(RUD_angle^2) | 0.015 | -0.003 | 0.033 | 0.009 | 0.09542 | . |
| qfac3:I(RUD_angle^2) | -0.004 | -0.022 | 0.014 | 0.009 | 0.65449 |  |
| qfac4:I(RUD_angle^2) | -0.017 | -0.034 | 0.001 | 0.009 | 0.06836 | . |
|  |  |  |  |  |  |  |
| Signif. codes: 0 ‘***’ 0.001 ‘**’ 0.01 ‘*’ 0.05 ‘.’ 0.1 ‘ ’ 1 | | | | | | |
|  |  |  |  |  |  |  |
| Residual standard error: 4.49 on 406 degrees of freedom | | | | | | |
| Multiple R-squared: 0.8003, Adjusted R-squared: 0.788 | | | | | | |
| F-statistic: 65.06 on 25 and 406 DF, p-value: < 2.2e-16 | | | | | | |
